# Case of Pemphigus

**Published:** 1807-07

**Authors:** T. M. Winterbottom

**Affiliations:** Physician to the Colony, at Sierra Leone


### *Case of Pemphigus.* By T. M. Winterbottom, M. D. *Physician to the Colony, at Sierra Leone.*

Mr. R. aged 63, tall and slender, and for many years past subject to complaints of the chest, threatening phthisis; was affected in the beginning of December, 1802, with great languor, lassitude, and loss of appetite. After these symptoms had continued with increasing debility for the space of a week, he complained of a violent itching and prickling in the soles of the feet, attended with so much soreness and pain as to prevent him from setting them on the ground. On the fourth day after thus complaining, a number of elevations, about the size of a pea, were observed on the soles of the feet; which continued to enlarge until they ran into each other, so that on the third morning, the whole of the cuticle was detached from the bottom of the feet, and resembled bladders distended with fluid. The cuticle being opened to relieve the painful distension, an almost colourless fluid was discharged; the skin, however, did not immediately collapse, but appeared as if filled with a gelatinous substance, from which a glutinous kind of fluid continued to distil. The whole of the internal surface seemed inflamed, of a fiery red colour, and was exquisitely sensible to the touch. During the three first days after the tumors were opened, the discharge was so profuse as to wet, not only the thick linen cloths which covered the feet, but also a blanket four times folded, placed underneath to preserve the bed, and which required to be changed twice in twenty-four hours. On the fourth day, the discharge began to abate, and continued gradually to diminish until the tenth, when it had nearly ceased. The collapsed cuticle had now become dry, and was beginning to separate in large portions, leaving underneath a delicate film, too sensible to bear the slightest pressure. The discharge from the feet, had a peculiarly offensive, nauseating smell; it scarcely tinged the linen, but rendered it as stiff as if it had been dipped in starch. The

The typhoid form of fever prevailed through the whole course of the complaint. The pulse, during the first three weeks, was generally about 110, and was remarkable for its softness, feebleness, and want of renitency. Neither thirst nor head-ach were much complained of, the heat of the skin was scarcely so great as in health. A low, muttering delirium, came on about the ninth day, and continued more or less severe, for the space of a month or five weeks, though for the last week or ten days it occurred chiefly after awaking from sleep. There was frequent sighing, and expression of general uneasiness and restlessness; indeed the characteristic feature of the disease was debility, which I never saw more strongly pourtrayed. A troublesome diarrhœa, sometimes attended with tenesmus, required the greatest care during the whole illness; two stools, in rather quick succession, invariably caused the pulse to flutter, and frequently produced fainting. A few grains of rhubarb were occasionally exhibited to remove the tenesmus, but the diarrhœa could only be checked by large doses of kino joined with a few drops of laudanum. The Peruvian bark appeared to be of the most essential service in supporting the strength: it did not seem to act upon the bowels, though during the space of six weeks, whilst the patient was considered in a hazardous state, from an ounce to ten drachms in powder, were daily swallowed. An anodyne was exhibited at night, with very good effect in abating the delirium. These means, and still more perhaps, the unremitting attention of his family in throwing in nourishment, very gradually restored him to his usual precarious state of health. No other application was made to the feet than a little mild ointment to prevent the adhesion of the cloths.

The following case of Pemphigus Apyretus occurred at the same time. Mrs. S. a maiden lady, aged 75, who had enjoyed almost complete immunity from disease, was suddenly affected with a sense of itching, burning pain in her legs, succeeded by superficial spots or efflorescences resembling flea-bites, which increased so much, as in twenty-four hours, to equal the size of a large hazel nut. These vesicles had a highly inflamed base; when burst, they poured out a thin yellowish fluid; and left superficial ulcerations, which continued acutely painful for some days, until they were covered with a dark coloured crust. No disorder of the system preceded these eruptions, nor did she make any other complaint, except of the pain, which she compared to the effects of boiling water. These eruptions eruptions continued to torment her for the space of two years, insomuch that she never had fewer upon her legs, the only part where they appeared, than six or eight in the different stages of their progress. Various internal remedies were used, among others, a slight mercurial course, but without effect. The only application which seemed to afford relief, was the ungt. flor. zinci; and this appeared to be merely from the ointment encrusting the sores, and protecting the painful ulceration beneath. The disposition to the disease appeared to wear gradually out, and for twelve months past, she has neither had any of the eruption, nor the slightest change in her usual good health.

